# Insights into the clinical features of thalassemia patients: a single-center study

**DOI:** 10.1097/MS9.0000000000004805

**Published:** 2026-03-03

**Authors:** Suad M. Jatal, Sawsan J. Harfouch, Rim M. Harfouch

**Affiliations:** a Faculty of Pharmacy, Alsham Private University (ASPU), Latakia, Syria; b Faculty of Pharmacy, Latakia (Tishreen) University, Latakia, Syria

**Keywords:** cross-sectional study, iron overload, Syria, Thalassemia

## Abstract

**Background:**

Thalassemia is a hereditary hemoglobinopathy that requires lifelong care, with β-thalassemia major being the most prevalent and severe form in the Mediterranean region, including Syria. Despite national programs for diagnosis and treatment, data on clinical patterns and healthcare challenges among Syrian patients remain scarce.

**Objective:**

This study aimed to characterize the demographic and clinical features of thalassemia patients in a city in Syria and identify gaps in management and preventive care.

**Methods:**

A cross-sectional study was conducted on 53 patients at the Thalassemia Center, between October 2024 and April 2025. Data were collected via patient interviews and a review of medical records. Variables included age, sex, thalassemia type, transfusion frequency, iron chelation therapy, splenectomy status, hemoglobin electrophoresis, ferritin levels, and vaccination status. Descriptive statistics and chi-square tests were used in the analysis.

**Results:**

The cohort comprised 28 males and 25 females, with β-thalassemia major accounting for 79.2% of cases and sickle cell thalassemia (15%). Elevated ferritin (>1000 ng/mL) was observed in 83% of patients, and 83% received iron chelation, mostly orally. Sickle hemoglobin was significantly associated with sickle cell thalassemia (*P* < 0.00001), and 77% had elevated fetal hemoglobin levels. Only 52.8% of the patients underwent splenectomy, and all required regular transfusions. Vaccination coverage varied: 77.3% received pneumococcal meningococcal vaccines, but only 11.3% received *Haemophilus influenzae* type B. Splenectomy was significantly associated with vaccine uptake (*P* = 0.016).

**Conclusions:**

Thalassemia patients exhibit high rates of iron overload and undervaccination, reflecting the challenges in disease monitoring and preventive care. Despite the availability of treatment, significant gaps remain in patient education, genetic counseling, and healthcare infrastructure. Strengthening multidisciplinary care, improving vaccination strategies, and enforcing genetic screening are essential for enhancing outcomes in this vulnerable population.

## Introduction

Thalassemia is a hereditary blood disorder resulting from mutations in globin genes, leading to reduced or absent synthesis of hemoglobin chains. β-Thalassemia, the most common and severe form, causes chronic anemia and requires lifelong management^[^1^]^.

β-Thalassemia is highly prevalent in the Mediterranean basin, Middle East, Africa, Southeast Asia, and Indian subcontinent. The spectrum of β-globin mutations varies significantly among ethnic groups and geographic regions. Hemoglobin disorders are particularly widespread in Syria owing to their strategic geographic location and genetic diversity^[^2^]^. A previous study found 17 different β-thalassemia mutations, accounting for almost 75% of the total β-thalassemia chromosomes in Syria^[^3^]^.

HIGHLIGHTS

- This cross-sectional study provides the first detailed clinical profile of thalassemia patients from a city in Syria.
- A high prevalence of β-thalassemia major (79.2%) and significant iron overload (ferritin >1000 ng/mL in 83%) was observed among patients.
- Only 52.8% of patients had undergone splenectomy, and vaccination coverage was suboptimal, especially for *Haemophilus influenzae* type B (11.3%).
- A statistically significant association was found between splenectomy and higher vaccine uptake (*P* = 0.016), highlighting gaps in preventive care.
- These findings underscore the urgent need to improve patient education, vaccination strategies, and genetic counseling in thalassemia management in low-resource settings.

β-Thalassemia is the most prevalent genetic disorder in Lebanon, as it is in many Mediterranean countries. Despite the implementation of premarital screening and public health efforts, sociocultural factors and conflict-related disruptions have impeded effective prevention, leading to continued births in affected children^[^4^]^.

The management of transfusion-dependent thalassemia relies heavily on regular blood transfusions to maintain hemoglobin levels above 9–10 g/dL, thereby preventing growth retardation and organ dysfunction. However, lifelong transfusions lead to iron overload, which can damage the heart, liver, and endocrine glands if not managed appropriately^[^5^]^. Iron chelation therapy, primarily with deferoxamine, deferiprone, or deferasirox, is essential for mitigating these effects^[^6^]^.

In Syria, treatment is provided through specialized thalassemia centers under the Ministry of Health. However, challenges, such as inconsistent drug supply, irregular monitoring, and limited access to MRI-based iron assessments, persist. A study by Yassouf *et al* highlighted poor compliance with deferoxamine therapy and associated thyroid dysfunction in Syrian patients, underscoring the consequences of inadequate long-term management. Strengthening patient education, improving drug availability, and enhancing monitoring infrastructure are crucial for better disease outcomes^[^7^]^.

Patients with thalassemia are at an increased risk of infection due to several factors, including frequent blood transfusions, iron overload, and splenectomy in many cases, which compromise the immune system. As a result, they require a broad range of vaccinations to protect against potentially serious infections such as hepatitis B, pneumococcal disease, *Haemophilus influenzae* type b, meningococcal infections, and seasonal influenza. These vaccines are essential to reduce morbidity and prevent life-threatening complications, as thalassemia patients may have a diminished ability to fight infections compared with the general population^[^8^]^.

Our study aimed to describe the clinical and demographic characteristics of thalassemia patients in a thalassemia center to provide essential epidemiological data from a region where such information is currently limited.

## Materials and methods

This cross-sectional study was conducted on 53 patients at a thalassemia treatment center between 30 October 2024, and 18 April 2025. The study was reported in accordance with the STROCSS 2021 guidelines^[^9^]^. Ethical approval for this study (Ethics Committee N° 369) was provided by the Ethics Committee of Alsham Private University, Latakia, Syria on 3 October 2024. Written informed consent was obtained from the patients or their parents/legal guardians prior to participation in the study.

Patient data were collected through direct interviews and a review of medical records. The information obtained included age, history of splenectomy, details of iron chelation therapy (type and method of administration), and whether a splenectomy had been performed. Additional data extracted from patient files included parental status (affected or carrier), number of blood transfusions, hemoglobin electrophoresis results, serum ferritin levels, and thalassemia type. Written informed consent was also obtained from the patients’ parents or legal guardians for publication and any accompanying images.

During the data collection period, complete blood count and differential tests were performed. Hemoglobin concentration and mean corpuscular volume (MCV) values were recorded based on these analyses. In addition, hemoglobin fraction analysis was performed using capillary electrophoresis, which had been previously conducted at the center using the Sebia Capillarys 2 Flex Piercing system (Sebia, France, 2020 model).

## Statistical analysis

All collected data were analyzed using Microsoft Excel. Descriptive statistical analyses of the patients’ demographic and clinical characteristics were performed. Chi-square tests and multivariate regression analyses were conducted using GraphPad Prism 10 (GraphPad Software, USA) to determine *P* values and statistically significant associations.

Continuous variables were summarized as mean ± standard deviation. The relationships between clinical parameters [blood transfusion rate, hemoglobin, and alanine aminotransferase (ALT)] and predictors (age, sex, and income) were assessed using linear regression. Age and income were treated as continuous variables, while sex was coded as male = 0 and female = 1. Normality of variables was checked using the Shapiro–Wilk test. Pearson correlation was used to assess the association between ALT and serum ferritin.

## Results

### Demographic characteristics

**Figure 1.**
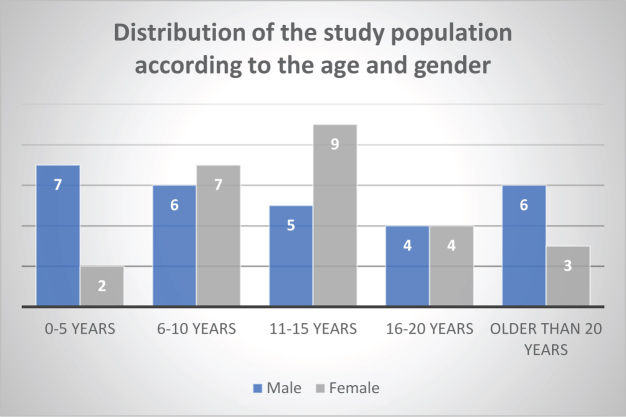
Distribution of thalassemia patients according to age groups and gender.

A total of 53 patients were enrolled in this study, consisting of 28 males (52.8%) and 25 females (47.2%). The median age was 12 years (IQR: 7–18). The age distribution revealed that the most represented age group was 11–15 years (43.4%), followed by the 6–10 years group (28.3%), as illustrated in Figure 1. This age distribution reflects the typical pediatric and adolescent burden of thalassemia observed in specialized treatment centers.

Regarding the type of thalassemia, β-thalassemia major (β-TM) was the predominant diagnosis, affecting 42 patients (79.2%). The gender distribution within this group was relatively balanced, with 52.4% being male and 47.6% female. A smaller subset of eight patients (15.1%) was diagnosed with sickle cell thalassemia, while the remaining patients had fewer common variants. This distribution aligns with the known epidemiological pattern of thalassemia in the region, where β-TM is more prevalent.

The analysis of parental carrier status revealed that more than half of the patients (54.7%) reported having a father who was a thalassemia carrier, while 52.8% reported that their mother was a carrier. Notably, a number of patients had both parents identified as carriers, indicating consanguinity or unrecognized risk factors. These findings underscore the importance of comprehensive family screening programs, community-level education, and the integration of genetic counseling services into thalassemia management protocols to help reduce disease incidence in future generations.

### Splenectomy status and blood transfusion

Linear regression analyses were conducted to assess the associations of age, sex, and income with blood transfusion. Blood transfusion rate was not significantly associated with age (slope = 0.00215, 95% CI: −0.0153 to 0.0196, *P* = 0.805) or sex (slope = 0.0199, 95% CI: −0.3004 to 0.3403, *P* = 0.901). However, it showed a significant negative association with income (slope = −0.00461, 95% CI: −0.00841 to −0.00081, *P* = 0.0184, *R*^2^ = 0.1043), indicating that patients with higher income required fewer transfusions (Table 1).

**Table 1:** Regression analysis of blood transfusion rate with age, sex, and income.

Predictor	Slope	Y-intercept	95% CI Y-intercept	Std. error Y-intercept	F (1,51)	*R*^2^	*P* value	Significant
Age	0.002154	1.499	1.211–1.786	0.1432	0.06175	0.001209	0.8047	No
Sex	0.01994	1.519	1.294–1.743	0.1118	0.01562	0.0003061	0.9010	No
Income	−0.004610	2.091	1.603–2.579	0.2431	5.937	0.1043	0.0184	Yes

**Figure 2.**
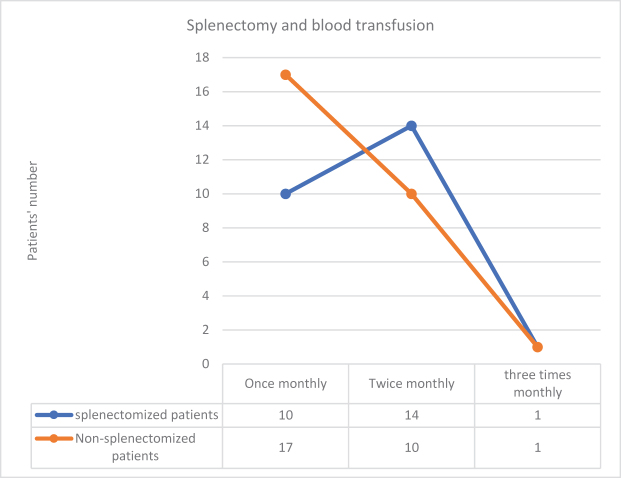
Blood transfusion according to splenectomy.

Regarding splenectomy, more than half of the patients were non-splenectomized and underwent blood transfusion in 28 of 53 patients (52.8%), and two-thirds of them underwent blood transfusion once monthly, as shown in Figure 2.

Using the chi-square test, the *P*-value was found to be 0.31, which was higher than 0.05, indicating that there was no statistically significant association between splenectomy and monthly frequency of blood transfusions.

### Liver enzymes functions

ALT was elevated (>45 U/mL) in 23 of 53 patients (43.4%), while aspartate aminotransferase was elevated in 24 of 53 patients (45.3%). ALT levels increased significantly with age (slope = 1.353, 95% CI: 0.459–2.246, *P* = 0.0037), indicating higher liver enzyme activity in older patients. No significant associations were found with sex (slope = 2.513, 95% CI: −15.34 to 20.36, *P* = 0.779) or income (*P* = 0.093), though a trend toward lower ALT values with higher income was observed (Table 1).

**Figure 3.**
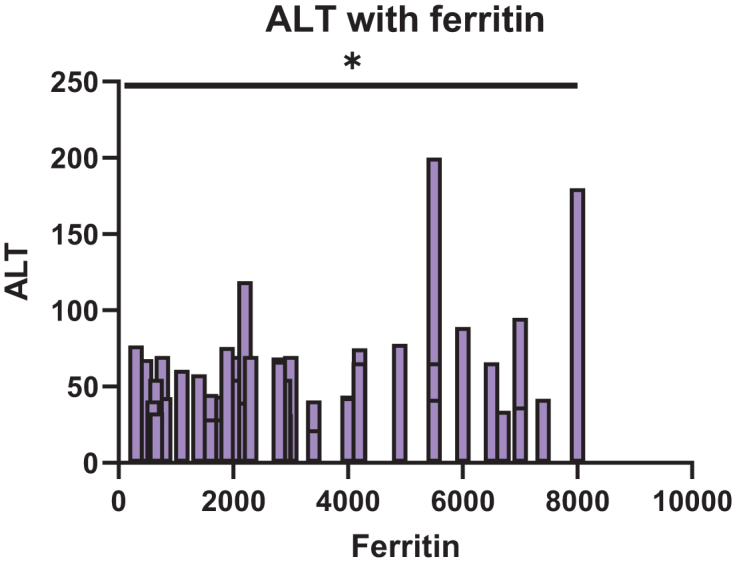
Correlation between ALT and ferritin levels across categories: <500, 500–999, 1000–2499, 2500–4999, and ≥5000 ng/mL (*P* = 0.0367).

A Pearson correlation analysis was performed to examine the relationship between ferritin and ALT levels in our 53 patients. Ferritin showed a significant positive correlation with ALT (*r* = 0.288, 95% CI: 0.019–0.518, *P* = 0.0367), indicating that higher ferritin levels were associated with higher ALT values as shown in Figure 3. The coefficient of determination (*R*^2^) was 0.083, suggesting that approximately 8.3% of the variability in ALT could be explained by ferritin levels.

### Iron chelator use and ferritin levels

Of the 53 patients, 44 (83%) were receiving iron chelation therapy. Most of them (73%) used oral chelators, while the remaining 27% received parenteral formulations. Similarly, 44 patients (83%) had ferritin levels exceeding 1000 ng/mL, well above the normal threshold of <500 ng/mL. In thalassemia, elevated ferritin typically reflects iron overload, particularly in transfusion-dependent individuals. This finding underscores the importance of regular monitoring and effective management to prevent long-term complications.

Linear regression analyses showed that ferritin levels increased significantly with age (slope = 88.44, 95% CI: 28.29–148.6, *P* = 0.0048), reflecting higher iron storage in older patients. No significant associations were observed with sex (slope = 20.80, 95% CI: −1177 to 1218, *P* = 0.972) or income (*P* = 0.091), although there was a slight trend toward lower ferritin levels in patients with higher income (Table 2).

**Table 2:** Regression analysis of hemoglobin levels with age, sex, and income.

Predictor	Slope	Y-intercept	95% CI Y-intercept	Std. error Y-intercept	F (1,51)	*R*^2^	*P* value	Significant
Age	88.44	2156	1162–3150	495.1	8.713	0.1459	0.0048	Yes
Sex	20.80	3360	2521–4199	417.8	0.001216	0.00002384	0.9723	No
Income	−12.52	4899	3026–6773	933.3	2.972	0.05506	0.0908	No

Analysis of the relationship between ferritin levels and MCV revealed a *P*-value of 0.053. Although this is slightly above the conventional significance threshold of 0.05, it suggests a marginal association between ferritin levels and MCV categories that were not statistically significant at the 5% level.

### Hemoglobin and hemoglobin fractions (HbA1, HbA2, HbF, and HbS)

Among 53 patients, 37 (69.8%) had hemoglobin levels below 8 g/dL, while 16 (30.2%) had levels between 8–12 g/dL. None had normal hemoglobin levels (>12 g/dL). Hemoglobin levels showed no significant associations with age (slope = 0.156, 95% CI: −0.978 to 1.289, *P* = 0.784), sex (slope = −0.954, 95% CI: −21.82 to 19.91, *P* = 0.927), or income (*P* = 0.471). *R*^2^ values were very low, indicating that these factors explained little of the variability in hemoglobin levels.

**Figure 4.**
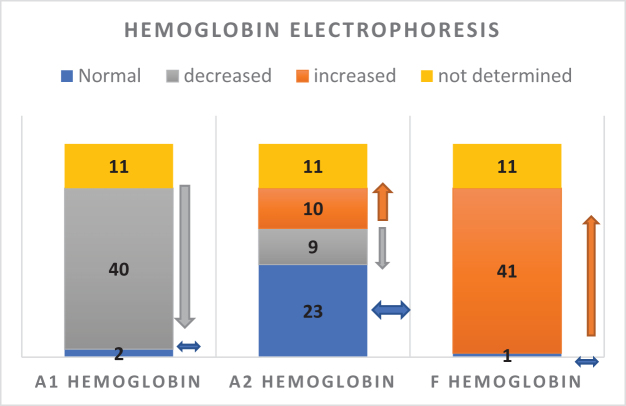
Hemoglobin electrophoresis results and distribution of hemoglobin types among patients.

In addition, 40 patients (75%) exhibited decreased hemoglobin A1 (HbA1), defined as less than 90% of the total hemoglobin. HbA2 levels were within the normal range in 23 patients (43%), elevated in 10 (19%), and below normal in 9 (17%; Fig. 4). Furthermore, 41 patients (77%) demonstrated elevated fetal hemoglobin (HbF) levels (>10%), far above the normal adult range of 0–1%. This likely reflects a compensatory response of erythropoietic tissues (bone marrow and liver) to enhance HbF production in the setting of impaired HbA1 synthesis.

Finally, 8 of 53 patients tested positive for sickle hemoglobin (HbS), while 45 tested negative. A chi-square analysis revealed a highly significant association between HbS positivity and thalassemia type (*P* ≈ 3.1 × 10^−12^), which aligns with the diagnostic role of HbS detection in sickle cell thalassemia.

### Vaccination status

**Figure 5.**
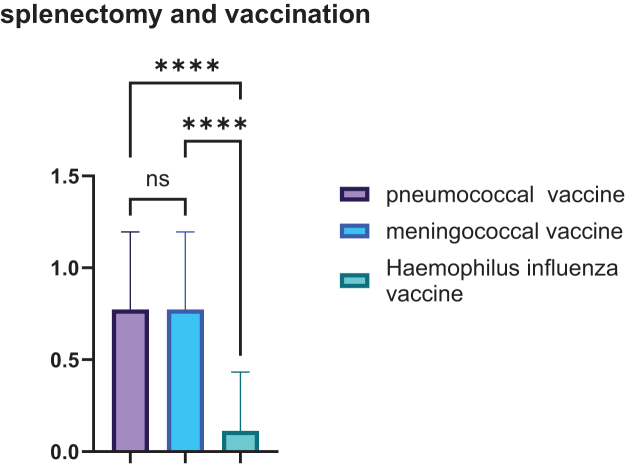
Correlation of splenectomy with vaccination status.

The majority of patients had taken both pneumococcal and meningococcal vaccines (41 of 53 patients, 77.3%), and only 6 of 53 patients (11.3%) received the Haemophilus influenza vaccine. We compared three vaccine groups – pneumococcal, meningococcal, and Haemophilus influenza, using one-way analysis of variance. The analysis showed a highly significant difference in group means [F(2,156) = 50.32, *P* < 0.0001, *R*^2^ = 0.392], meaning that about 39% of the variation in values was explained by the type of vaccine. Tests for equal variances confirmed that the groups had similar variability (*P* = 0.084). Post-hoc Tukey comparisons revealed that the Haemophilus influenza group had significantly lower values than both the pneumococcal (mean difference = 0.660, 95% CI: 0.481–0.840, *P* < 0.0001) and meningococcal groups (mean difference = 0.660, 95% CI: 0.481–0.840, *P* < 0.0001), while pneumococcal and meningococcal groups were similar to each other (mean difference = 0.000, 95% CI: −0.180–0.180, *P* > 0.9999), as shown in Figure 5.

## Discussion

This study offers important insights into the clinical and demographic characteristics of patients with thalassemia in a city in Syria, highlighting the common features and unique challenges faced by this population. Our findings align with existing regional and international data while also revealing critical areas in need of attention to improve patient outcomes.

The demographic distribution of patients showed a nearly equal sex ratio between males and females, consistent with the autosomal recessive inheritance pattern of thalassemia^[^10^]^. The predominance of β-TM (79.2%) among our study population is in line with previous reports from Syria and other Mediterranean countries, where this form of the disease is most common, according to previous published paper, β-TM represented 73.9% of all types of thalassemia in Iraq^[^11^]^. A significant proportion of patients with sickle cell thalassemia (15%) underscored the genetic diversity of hemoglobinopathies in the region. This diversity is likely influenced by historical migration, consanguinity, and the molecular profile of hemoglobinopathies, which are common genetic disorders among Arabs, with reported carrier rates for the sickle cell trait ranging from 0.3% to 30%^[^12^]^.

Parental carrier status data reinforce the urgent need for public health strategies that focus on education and genetic counseling. The case of parents who are both affected by thalassemia and still choose to conceive a child illustrates the critical gaps in awareness and the necessity of enforcing preventive measures such as premarital screening and community education^[^13^]^.

Clinical characteristics revealed several important patterns. Over half of the patients had not undergone splenectomy yet required regular transfusions, reflecting an early stage of disease management or decisions based on clinical indications. Our analysis found no statistically significant association between splenectomy and the frequency of transfusions, suggesting that other factors such as individual disease severity or treatment protocols may play a more decisive role^[^14^]^.

Iron overload remains a major concern, with 83% of patients in our cohort exhibiting ferritin levels >1000 ng/mL – a predictable yet worrisome consequence of chronic transfusions. Sustained ferritin levels above 2500 ng/mL are known to increase the risk of cardiac toxicity and death in thalassemia patients^[^15^]^. The high reliance on oral chelators (73%) suggests a shift toward more patient-friendly regimens; however, their effectiveness requires continuous monitoring, particularly in settings with limited access to MRI-based assessments^[^16^]^. By comparison, a recent Saudi Arabian cohort reported that most patients had ferritin levels >2500 ng/mL, with a mean of 3964.5 ± 1968 ng/mL despite chelation therapy, highlighting an even greater burden of iron overload. This underscores regional variability in disease control and the persistent challenges of managing iron toxicity across different healthcare systems^[^17^]^. Furthermore, studies have documented geographical differences in iron overload prevalence: cardiac siderosis affects over 25% of thalassemia major patients in Southeast Asia but only 15–20% in Europe and the Middle East, reflecting disparities in chelation practices and access to advanced monitoring technologies^[^18^]^.

Although most of our patients had ferritin levels >1000 ng/mL, we were unable to systematically assess organ-specific complications of iron overload, such as cardiac dysfunction (via echocardiography or T2* MRI), hepatic injury (through serial liver enzymes or fibrosis markers), or endocrine disturbances (hormone profiles). This absence of detailed organ evaluation is a significant limitation of the study and reflects the restricted availability of advanced diagnostic tools in low-resource settings. Future multicenter research incorporating cardiac, hepatic, and endocrine assessments is needed to provide a more comprehensive understanding of iron-related morbidity in Syrian thalassemia patients.

Hemoglobin electrophoresis results confirmed the expected diagnostic patterns, with HbS positivity showing a highly significant association with sickle cell thalassemia (*P* < 0.00001). The widespread elevation of HbF observed in our cohort is consistent with its known role in mitigating disease severity and reflects a compensatory response to impaired HbA synthesis. In the absence or reduction of β-globin production, the body upregulates γ-globin synthesis, which combines with the α-globin chains to form HbF (α₂γ₂). This adaptive mechanism helps sustain hemoglobin production and preserves the oxygen-carrying capacity of affected individuals^[^19^]^.

Vaccination coverage varied widely, with pneumococcal and meningococcal vaccines reaching 77.3% uptake. However, *H. influenzae* type b coverage was notably lower (11.3%). Importantly, a statistically significant association (*P* = 0.016) was found between splenectomy and vaccine uptake, suggesting that clinical guidelines recommending post-splenectomy vaccinations are being followed more rigorously in this subgroup^[^20,21^]^. Nonetheless, efforts should be made to ensure broader immunization coverage. The very low Hib coverage we observed contrasts sharply with best-practice recommendations for asplenic/hyposplenic patients (Hib, pneumococcal, meningococcal, and influenza), and with Saudi surgical series where pneumococcal vaccination before splenectomy was near-universal^[^22^]^. Possible reasons for the low Hib coverage in our setting include vaccine costs not being consistently subsidized, disruption of immunization programs during years of armed conflict, limited availability of specific vaccines in local markets, and broader economic constraints affecting patient access to preventive care.

A key strength of this study is that it provides a detailed clinical and demographic overview of thalassemia patients in a Syrian treatment center, a population for which current data are scarce. The inclusion of both β-TM and sickle cell thalassemia patients allows for broader insights into regional hemoglobinopathy patterns. The use of both patient interviews and medical record reviews ensured comprehensive data collection, and the statistical analysis helped identify clinically relevant associations.

However, the study has several limitations. The single-center design and modest sample size may limit the generalizability of these findings to other regions. Larger multicenter studies are warranted to validate these observations. Additionally, the cross-sectional nature of the study precludes assessment of long-term outcomes. Some variables, such as compliance with chelation therapy or socioeconomic factors influencing vaccination, were not explored in depth, which could have provided more contexts for the observed trends. Although ALT values were available and analyzed in relation to ferritin, other key assessments such as echocardiography, detailed liver panels, and endocrine hormone levels were not consistently available. This limited our ability to directly link iron burden to specific organ damage and represents a major limitation of the study.

In conclusion, patients with thalassemia face challenges typical of low-resource settings including limited access to comprehensive care and preventive education. Despite these hurdles, many patients are receiving critical components of standard treatment, such as iron chelation and vaccination. Strengthening the healthcare infrastructure, ensuring regular monitoring, and enhancing public awareness are essential steps to improve health outcomes for this vulnerable population. In practical terms, our findings support several specific recommendations: implementing national vaccination strategies to improve Hib coverage; expanding genetic counseling and premarital screening programs; ensuring consistent supply of, and adherence to, iron chelation therapy; and improving access to diagnostic tools such as echocardiography, liver panels, and MRI T2* for early detection of organ damage.

## Data Availability

The data that support the findings of this study are available from the corresponding author upon reasonable request. Any shared data will be de-identified to protect patient confidentiality.

## Research registration unique identifying number (UIN)

This retrospective study was based on analysis of previously collected, anonymized data. No new patient recruitment or intervention occurred. As such, registration in a clinical trial registry was not applicable. The study was conducted in accordance with the Declaration of Helsinki.
